# Onychomadesis and potential association with HFMD outbreak in a kindergarten in Hubei province, China, 2017

**DOI:** 10.1186/s12879-019-4560-8

**Published:** 2019-11-26

**Authors:** Dan Li, Yang Wu, Xuesen Xing, Jigui Huang, Anlu Mao, Tian Liu, Ping Rao, Wei Qin, Lijie Zhang, Luzhao Feng, Shangren Gao, Xuhua Guan

**Affiliations:** 1 Division of Infectious Disease, Jingmen Center for Disease Control and Prevention, Jingmen, 448000 Hubei China; 2 Division of Infectious Disease, Hubei Provincial Center for Disease Control and Prevention, Wuhan, 430079 China; 3 Chinese Field Epidemiology Training Program, Beijing, 100050 China; 4 Division of Infectious Disease, Jingzhou Center Disease Control and Prevention, Jingzhou, 434000 Hubei China; 5 Jingmen Municipal Commission of Health and Family Planning, Jingmen, 448000 Hubei China; 6 Lu’an Center Disease Control and Prevention, Lu’an, 237008 Anhui China; 7 Division of Infectious Disease, Chinese Center for Disease Control and Prevention, Key Laboratory of Surveillance and Early-warning on Infectious Disease, Beijing, 102206 China

**Keywords:** Onychomadesis, Hand, Food and mouth disease, Outbreak

## Abstract

**Background:**

In 2017, an outbreak of onychomadesis occurred in kindergarten H, Hubei province, China. We investigated the field to learn about the magnitude and reason of the outbreak.

**Methods:**

The case definition was that a child with onychomadesis or transverse ridging (Beau’s line) in fingernails and toenails without previous traumatic or systemic disease in kindergarten H from Sep. 1st to Nov. 30th, 2017. A retrospective cohort study was carried out to analyze the epidemiological relationship between onychomadesis and the hand-foot-mouth disease (HFMD) in Primary Class #2, kindergarten H. We also performed a serological survey for neutralizing antibodies against coxsackie virus A6 (CVA6), coxsackie virus A10 (CVA10) among 15 cases and six healthy children in the kindergarten. Meanwhile, some children were carried out with routine blood, fungal microscopic and microelement tests. Indoor environment examinations had been done for all classes.

**Results:**

A total of 20 cases were identified in Kindergarten H. Seventy-five percent (15/20) cases occurred in Primary Class #2. Fifty-five percent of the cases (11/20) had suffered from HFMD within two months. The median time between onychomadesis and HFMD was 45 days (ranging from 31 to 58 days). A retrospective cohort study in Primary Class #2 showed the attack rate was 90.0% among 10 children who suffered from HFMD in the past two months compared to 30.0% among 20 children who didn’t (Rate Ratio [RR] =3.0, 95% Confidence Interval [CI] =1.5–6.0). The positive rates of neutralizing antibodies were 66.7% for CVA6 and 26.7% for CVA10 in tested cases. The result of routine blood, fungal microscopic, microelements tests were normal in cases. The indicators of environment were within the normal range.

**Conclusion:**

The results of this study suggested that the outbreak of onychomadesis in Hubei province was probably associated with HFMD epidemic within two months.

## Background

Onychomadesis is the shedding of the nails beginning at the proximal end, possibly caused by the temporary arrest of the function of the nail matrix, and can affect both fingernails and toenails [1, 2]. Nail matrix arrest may result in a variety of changes, including nail shedding (onychomadesis) and transverse ridging (Beau’s lines) [3]. It is a rare disorder in children, and cases were considered to be idiopathic or acquired [3]. HFDM viruses are potential risk factors of onychomadesis in children [4–19]. Onychomadesis after HFMD was first reported in five children in Chicago, USA, 2000 [4]. A similar report of four children with onychomadesis published in Liège, Belgium, 2001 [5]. Onychomadesis outbreaks associated with HFMD had also been reported in Spain [3, 6–11], Finland [12, 13], Taiwan [14], Greece [15], Japan [16]. In the mainland China, an onychomadesis outbreak after HFMD occured in Hangzhou in 2016 [17], and 43.1% HFMD cases suffered from onychomadesis between the 3th and 8th week after onset in an HFMD outbreak in Beijing, 2015 [18]. Molecular characterization of the etiologic agent involved in onychomadesis after HFMD, remains controversial [19]. Onychomadesis has a strong association with CVA6 and CVA10, and other serotypes such as coxsackie virus B1 (CVB1), coxsackie virus B2 (CVB2), coxsackie virus A5 (CVA5), enterovirus 71 (EV71), coxsackie virus A16 (CVA16), enterovirus 9 (E9) [7–19]. HFMD is a common viral illness that usually affects infants and children younger than five years old. The main symptoms are fever, sore throat, general malaise, and vesicular eruptions on the palms of the hands, oral mucosa, soles of the feet, and tongue [10]. 7,200,092 probable cases of HFMD were reported in notifiable disease monitoring system of China during 2008–2012, and EV71 predominated in laboratory-confirmed cases, which accounted for 93% of fatal cases, 80% of severe cases, and 45% of mild cases [20]. EV71, CVA16, and other enteroviruses co-circulated during 2008–2012 in China [20–22]. The proportions were 45% for other enteroviruses, 41% for EV71, and 14% for CVA16 in laboratory-confirmed HFMD cases in China in 2017, while the proportions were 76% for other enteroviruses, 10% for EV71, and 15% for CVA16 in Hubei province in 2017 according to the notifiable disease monitoring system of China. Eleven children suffered from onychomadesis were reported to Hubei provincial center for disease control and prevention in the same class in Kindergarten H in Songzi County on Nov.21th, 2017. We immediately conducted the investigation to find out the magnitude and potential reason of this onychomadesis outbreak.

## Methods

### Epidemiological investigations

A case was defined as a child with onychomadesis or Beau’s line in fingernails and toenails without previous traumatic or systemic disease in Kindergarten H, Songzi County, Hubei province from Sep. 1st to Nov. 30th, 2017. The diagnosis was conducted through on-site checking of nails by pediatricians and dermatologists, combined with interviewing kindergarten staffs, and reviewing routine health and absence records of children. Questionnaires were used to collect the information about the onset, diagnosis and treatment, illness in the last two months, behavior habits and nutrition condition. Meanwhile, we asked if similar cases had occurred in four nearby kindergartens in the county. A retrospective cohort study was under-taken to analyze the relationship between onychomadesis and the prevalence of HFMD in recent two months in Primary Class #2 in Kindergarten H. EXCEL2007 was used to collate data and draw the epidemic curve, and SPSS13.0 was used to analyze the data.

### Laboratory tests

Blood samples of six cases and six healthy children were collected to identify their microelement status including calcium, iron, zinc, lead, cadmium, copper and magnesium. The indoor environment of all classes in Kindergarten H was examined with the indicators of formaldehyde, benzene, toluene, and xylene. Six pieces of plasticines used by cases were detected for the lead. Fungal microscopic examinations of nail smear samples were taken. We collected the anal swabs and serum specimens from 15 cases and 6 healthy children. Viral RNA was extracted using a QIAamp Viral RNA Mini Kit (Qiagen, Valencia, CA, USA). We tested RNA from each sample for sequencing VP1 (viral protein 1) gene of enterovirus including EV71, CA16, CA6 and CA10 by real-time reverse transcriptase-polymerase chain reaction (RT-PCR) using fluorescent PCR detection kits for HFMD viral RNA (Shuoshi Biotechnology company, Jiangsu, China). Neutralizing antibody tests of CA6 and CA10 were performed for serum specimens as described [23]. A neutralizing titer of≥1:16 was considered as a threshold for positivity [23, 24].

## Results

### Descriptive epidemiology

**Fig. 1:**
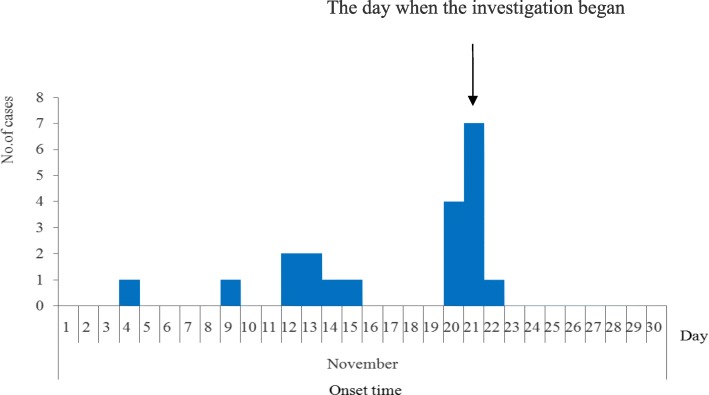
The epidemic curve of the onychomadesis outbreak in Kindergarten H, Hubei province, 2017

**Fig. 2:**
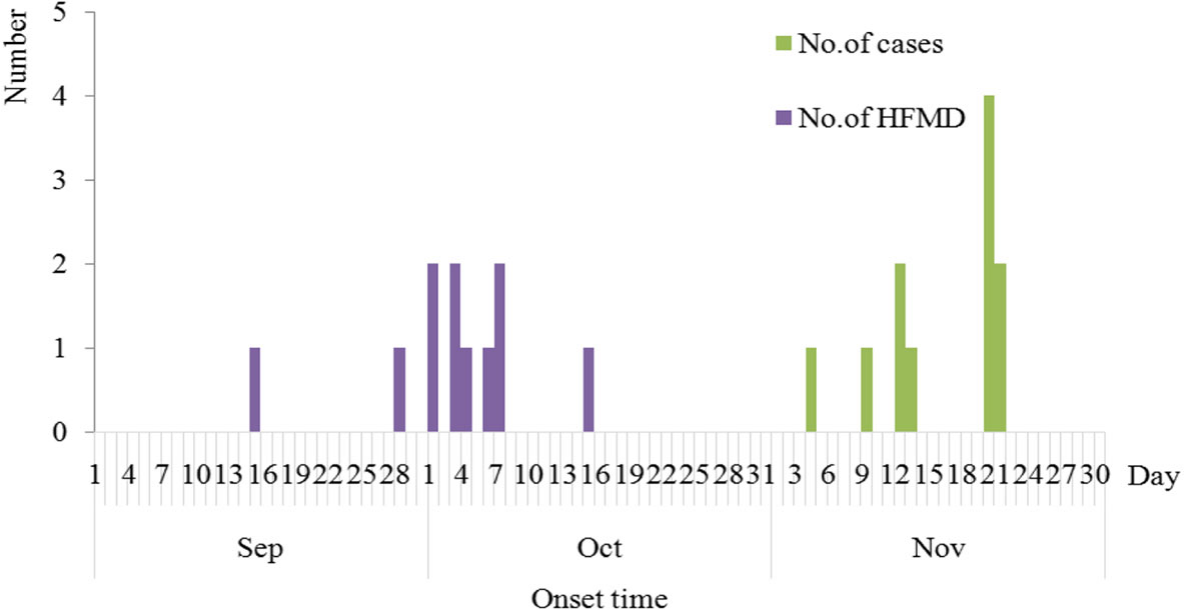
The onset time of the onychomadesis and HFMD from Sep to Nov in Kindergarten H, Hubei province, 2017

**Fig. 3:**
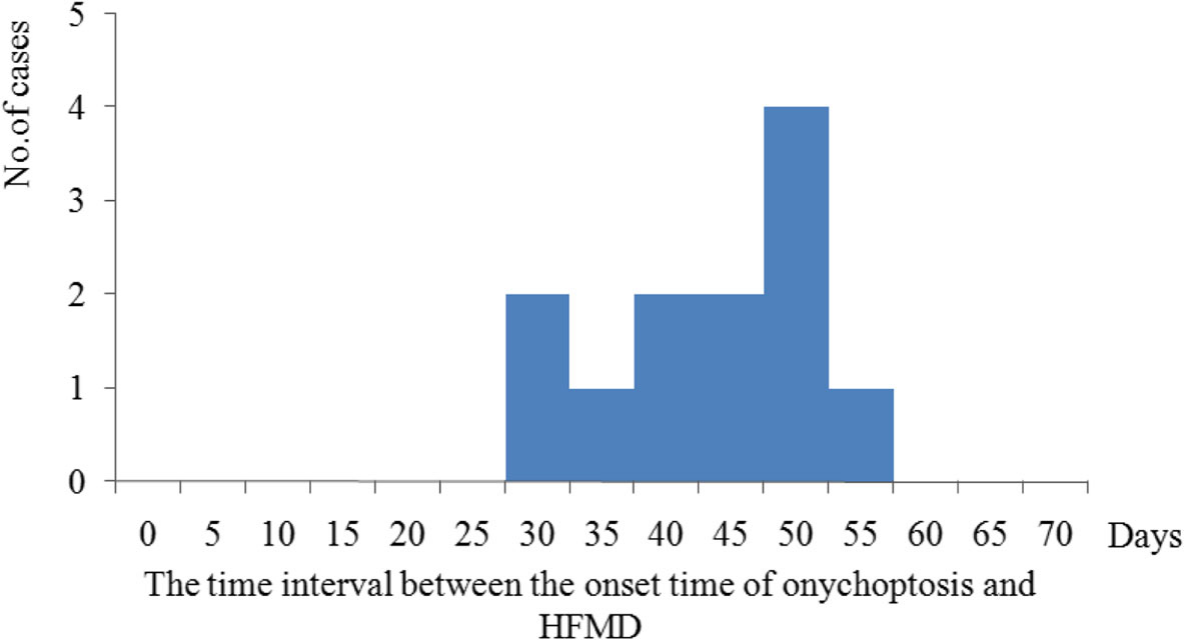
The time interval between the onset time of onychomadesis and HFMD in Kindergarten H, Hubei province, 2017

A total of 20 cases were identified in Kindergarten H which has 250 children in our study. The attack rate was 8.0% (20/250), and there was no severe or death case. All 20 cases (100.0%) has occurred onychomadesis and 4 cases (20.0%) also had Beau’s line in their nails. Onychomadesis started from the proximal deck and gradually developed to the distal deck. The numbers of affected fingers or toes were between one and nine with the average two. Eighteen and two cases had unhealthy fingernails and toenails respectively, while two cases had unhealthy fingernails and toenails simultaneously. The onychomadesis cases occurred between Nov. 4th and Nov. 22th as shown in Fig. 1. The attack rates of male and female children in kindergartens were 6.7% (8/118) and 9.1% (12/132) respectively, without statistical difference (*x*^2^ = 0.45, *P* > 0.05). The median age of the 20 patients was 3 years old (range, 3–4 years). There were seven classes in kindergarten H, and three of them had cases with onychomadesis. 75.0% (15/20) cases occurred in Primary Class #2. According to the retrospective interview, 55.0% of the cases (11/20) had suffered from HFMD characterized by fever (72.8%) and vesicular eruptions (100.0%) on hands or feet or in the mouth, within two months before the onset of onychomadesis. The median time between onychomadesis and HFMD was 45 days (ranging from 31 to 58 days) (Figs. 2, 3).

### Analysis of risk factors

A retrospective cohort study in Primary Class #2 showed the attack rate was 90.0% among ten who suffered from HFMD in recent two months compared to 30.0% among 20 who didn’t (Rate Ratio [RR] = 3.0, 95% Confidence Interval [CI] = 1.5–6.0) (Table 1).

**Table 1:** Analysis of risk factor of onychomadesis in Primary Class #2 of Kindergarten H, Hubei province, 2017

Factor: Suffered from HFMD in the past two months	No. of cases	Overall No.	Attack rate (%)	*RR* (95% *CI*)
Yes	9	10	90.0	3.0 (1.5–6.0)
No	6	20	30.0	Ref.

### Laboratory test results

The positive rates of neutralizing antibodies were 66.7% (10/15) for CVA6 and 26.7% (4/15) for CVA10 in tested cases. 66.7% (10/15) of tested cases and 100% (6/6) tested healthy children were positive for CVA6 or CAV10 neutralization antibody test, without statistical difference (Fisher’s exact probability method, *P* > 0.05) (Table 2). All anal swabs samples were negative for HFMD nucleic acid test. Indicators of microelement, routine blood, fungal microscopic and environment of tested cases were within the normal range (Additional file 1 Table S1).

**Table 2:** Results of HFMD neutralization antibody test in Kindergarten H, Hubei province, 2017

Source of specimens	No. of specimens	Positive No. for neutralization antibody test		
		CVA6	CVA10	CVA6 or CVA10
Cases	15	10	4	10
Healthy children	6	4	3	6

## Discussion

8.0% (20/250) of children suffered from onychomadesis in kindergarten H, Songzi county, Hubei province, from Sep 1 to Nov 30 in 2017. Fifty-five percent of the cases (11/20) had suffered from HFMD within two months before onychomadesis. The median time between onychomadesis and HFMD was 45 days (ranging from 31 to 58 days). A retrospective cohort study in primary class #2 showed that children who had suffered from HFMD within two months had three times of the risk of onychomadesis. The positive rates of neutralizing antibodies were 66.7% for CVA6 and 50.0% for CVA10. The results of this study suggested that the onychomadesis epidemic occurred in Hubei province was probably associated with HFMD within two months prior to the onset of the disease. In our study, the factors of idiopathic, medicine, fungal microscopic, microelement, and environment for onychomadesis were almost excluded. The symptoms of cases were similar with other onychomadesis outbreak after HFMD (Table 3) [3–18]. The median time between onychomadesis and HFMD in this outbreak was 45 days (ranging from 31 to 58 days), which was also similar with other references except for a little shorter than 36–69 days in an onychomadesis outbreak, Spain, 2009 [7]. 66.7% (10/15) of the cases were positive for CVA6 or CAV10 neutralization test, which was similar with 65.9% (29/44) positive in an onychomadesis outbreak, Spain, 2008 [9]

**Table 3:** Reported outbreaks of onychomadesis associated with HFMD

Study	Title	District
Clementz GC et al. 2000 [4].	Nail matrix arrest following hand–foot–mouth disease: a report of five children.	Chicago, USA
Bernier V et al. 2001 [5].	Nail matrix arrest in the course of hand, foot and mouth disease.	Liège, Belgium; Bordeaux, France
Salazar A et al. 2008 [3].	Onychomadesis outbreak in Valencia, Spain, June 2008.	Valencia, Spain
Redondo Granado MJ et al. 2009 [6].	Brote de onicomadesis posvírica en Valladolid.	Valladolid, Spain
Osterback R et al. 2009 [12].	Coxsackievirus A6 and hand, foot, and mouth disease, Finland.	Finland
Blomqvist S et al. 2010 [13].	Co-circulation of coxsackieviruses A6 and A10 in hand, foot and mouth disease outbreak in Finland.	Finland
Cabrerizo M et al. 2009 [7].	Onychomadesis after a hand, foot, and mouth disease outbreak in Spain, 2009.	La Coruna, Spain
Guimbao J et al. 2010 [8].	Onychomadesis outbreak linked to hand, foot, and mouth disease, Spain, July 2008.	Saragossa, Spain
Davia JL et al. 2011 [9].	Onychomadesis outbreak in Valencia, Spain associated with hand, foot, and mouth disease caused by enteroviruses.	Valencia, Spain
Bracho MA et al. 2011 [10].	Enterovirus co-infections and onychomadesis after hand, foot, and mouth disease, Spain, 2008.	Valencia, Spain
Wei SH et al. 2011 [14].	An outbreak of coxsackievirus A6 hand, foot, and mouth disease associated with onychomadesis in Taiwan, 2010.	Taiwan, China
Navarro Moreno E et al. 2014 [11].	Outbreak of hand, foot and mouth disease with onychomadesis caused by Coxsackie virus A16 in Granada.	Granada, Spain
Miyamoto A et al. 2014 [16].	An outbreak of hand-foot-and-mouth diseasemimicking chicken pox, with a frequent association of onychomadesis in Japan in 2009: a new phenotype caused by coxsackievirus A6.	Oita, Japan
Apalla Z et al. 2015 [15].	Onychomadesis after hand-foot-and-mouth diseaseoutbreak in northern Greece: case series and brief review of the literature.	Northern Greece
Kao QJ et al. 2016 [17].	An outbreak of coxsackievirus A6 hand, foot, and mouth disease associated with onychomadesis in Hangzhou.	Hangzhou, China
Li J et al. 2018 [18].	An outbreak of Coxsackievirus A6-associated hand, foot, and mouth disease in a kindergarten in Beijing in 2015.	Beijing, China

The positive rate of neutralizing antibodies was 58% for CVA6 and 43% for CVA10 in healthy children aged 4–6, China [24]. In our study, the positive rate of neutralizing antibodies was 66.7% for CVA6 and 26.7% for CVA10 in cases, while 66.7% for CVA6 and 50.0% for CVA10 in healthy children. The high positively rates probably caused by the concentration of Hand-foot-and-mouth disease within two months before the onset of the disease, which also supported our conclusion. Reports about the mechanism of onychomadesis after HFMD were limited. Onychomadesis may occur after inflammation of the nail matrix or maceration associated finger blisters because of traumatic nail loss [19]. The shed nail fragments of a patient who suffered onychomadesis after HFMD were positive for CVA6 nucleic acid test in an outbreak of onychomadesis, which suggested that CVA6 virus replication caused the damage of nail matrix, resulting in onychomadesis [12]. HFMD outbreak frequently occurs in kindergarten, but there were few reports of onychomadesis outbreak in China [17, 18]. The pathogens of the HFMD kept drifting in different countries [25–30]. The EV71 inactivated vaccine successfully entered the market in China since June 2016 [31]. Other enterovirus (except EV71 and CVA16) in the confirmed cases of HFMD were the predominate pathogen (accounted for 76%) in Hubei province in 2017, which was different from the past pathogen trends and showed the change of major pathogen [20, 21]. An outbreak of HFMD in 2016 showed that the positive rate was 76% (146/192) for CVA6 and 2.6% for CVA10 in Chongqing [32]. In our study, the HFMD outbreak before onychomadesis outbreak only affected Primary Class #2 by the passive and active surveillance in Kindergarten H, which may related to the contact transmission. Our study is the first reported onychomadesis outbreak in Hubei province and it was rare in China, which means further research is necessary to learn about its etiology, epidemic trend, prevention and control, and the relationship with onychomadesis. There are several limitations in our study. The positive rate of HFMD nucleic acid test was low, and the results of neutralizing antibody test only suggested previous infection. The HFMD nucleic acid test was delayed and we collected anal swabs rather than feces or shed nail described in previous literatures [10, 12, 13]. The results of zero prevalence of HFMD virus in anal swabs by using nucleic acid tests were predictable regarding the clearance of HFMD virus after recovery. In addition, we only tested the neutralizing antibodies CVA6 and CVA10 which were common pathogens related to onychomadesis. The emergency stocks of the neutralizing antibodies for HFMD were insufficient. The surveyed pathogenic types of HFMD were EV71, CVA16 and other enteroviruses, and further classification of other enteroviruses was not official requirement yet in Hubei province.

## Conclusions

In conclusion, the first onychomadesis outbreak occurred in Hubei province was probably associated with HFMD epidemic within two months after onset.

## Supplementary information

**Additional file 1: Table S1.** Results of blood and environment tests in Kindergarten H, Hubei province, 2017.

## Data Availability

The datasets used and/or analysed during the current study available from the corresponding author on reasonable request.

## Abbreviations

CDC: Center for Disease Control and Prevention
CI: Confidence Interval
CVA10: Coxsackie virus A10
CVA16: Coxsackie virus A16
CVA5: Coxsackie virus A5
CVA6: Coxsackie virus A6
CVB1: Coxsackie virus B1
CVB2: Coxsackie virus B2
E9: Enterovirus 9
EV71: Enterovirus71
HFMD: Hand-foot-mouth disease
RT-PCR: reverse transcriptase-polymerase chain reaction
VP1: Viral protein 1; RR: Rate Ratio
