# Supplementary material for: Onychomadesis and potential association with HFMD outbreak in a kindergarten in Hubei province, China, 2017
Source: BMC Infect Dis. 2019 Nov 26;19:995. doi: 10.1186/s12879-019-4560-8 (PMC6878681; doi:10.1186/s12879-019-4560-8)
Supplement: Supplementary file 1 — Additional file 1: Table S1. Results of blood and environment tests in Kindergarten H, Hubei province, 2017. [file 12879_2019_4560_MOESM1_ESM.docx]

**Additional file: Table**

**Supplementary data**

Supplementary Table 1 The results of blood and environment tests in Kindergarten H, Hubei province, 2017.

| Items* | Objectives | Results |
| --- | --- | --- |
| Microelement | Six cases in small class two | Normal |
| Microelement | Six healthy children in small class two | one children with mildly calcium deficient and one children with iron deficient |
| Routine fungal microscopic examinations | Ten cases in small class two | Normal |
| Routine blood | Ten cases in small class two | Normal |
| Lead | six pieces of [plasticine](javascript:;)s used by cases | Normal |
| Formaldehyde, benzene, toluene and xylene | The indoor environment of all classes | Normal |

***All of the testing items were carried out according national standards.**
